# The Effectiveness of Transcranial Magnetic Stimulation in Adolescents and Young Adults With Major Depressive Disorder

**DOI:** 10.1016/j.jaacop.2025.06.006

**Published:** 2025-07-01

**Authors:** Paul E. Croarkin, Scott T. Aaronson, Linda L. Carpenter, Todd M. Hutton, Kenneth Pages, Bing Chen, Harold A. Sackeim

**Affiliations:** a Mayo Clinic, Rochester, Minnesota; b Sheppard Pratt Health System and University of Maryland, Baltimore, Maryland; c Butler Hospital and the Warren Alpert Medical School of Brown University, Providence, Rhode Island; d Southern California TMS Center, Los Angeles, California; e TMS of South Tampa, Tampa, Florida; f NAMSA, St. Louis Park, Minneapolis, Minnesota; g Medical University of South Carolina, Charleston, South Carolina

**Keywords:** adolescence, dose-response, major depressive disorder, patient registry, transcranial magnetic stimulation (TMS)

## Abstract

**Objective:**

To assess the real-world effectiveness of transcranial magnetic stimulation (TMS) for depression in large adolescent and young adult samples.

**Method:**

Clinical outcome data from 364 sites were extracted for adolescent (n = 682; ages 12-19 years) and young adult (n = 601; ages 20-21 years) patients treated with TMS for major depressive disorder. Primary outcomes included response and remission rates assessed with the Patient Health Questionnaire-9 (PHQ-9). Secondary outcomes included rates of clinically meaningful benefit and worsening of depression based on a meaningful change threshold of ±6 points on the PHQ-9, dose-response relationships in effectiveness, and trajectories of symptomatic improvement over the course of TMS treatment.

**Results:**

PHQ-9 response and remission rates were equivalent in adolescent and young adult groups. Following the TMS course, approximately 70% of both groups reported meaningful improvement, whereas less than 1% reported meaningful worsening. There were marked dose-response effects, with longer courses of TMS associated with greater improvement (*F*_5,1277_ = 19.10, *p* < .0001). The trajectory of improvement showed greatest symptom reduction over the first 10 sessions and with steady improvement thereafter. The adolescent and young adult groups did not differ in these outcomes, and their patterns mirrored those reported in a large adult registry sample.

**Conclusion:**

This study examined the largest samples to date of adolescents and young adults treated with TMS for major depressive disorder. In both groups, TMS resulted in marked improvement in depressive symptom severity. The magnitude of benefit, trajectory of symptomatic improvement, and dependency on number of treatment sessions demonstrated therapeutic effects similar to that reported in adults.

**Study Registration Information:**

A Retrospective Study to Evaluate NeuroStar Advanced Therapy in Adolescents; https://clinicaltrials.gov/study/NCT06699940

**Diversity & Inclusion Statement:**

We worked to ensure sex and gender balance in the recruitment of human participants. We actively worked to promote sex and gender balance in our author group. We actively worked to promote inclusion of historically underrepresented racial and/or ethnic groups in science in our author group. While citing references scientifically relevant for this work, we also actively worked to promote sex and gender balance in our reference list. While citing references scientifically relevant for this work, we also actively worked to promote inclusion of historically underrepresented racial and/or ethnic groups in science in our reference list.

## Plain language summary

This study examined the effectiveness of Transcranial Magnetic Stimulation (TMS) for the treatment of major depressive disorder in 682 adolescents and 601 young adults. In both groups, TMS resulted in marked improvement in depressive symptom severity, with nearly 70% reporting meaningful improvement. The overall effectiveness of TMS, the dose-response relationship with the number of sessions, and the trajectory of improvement over the TMS course were similar in the adolescent and young adult groups and mirrored the same effects reported in adults.

Each year, approximately 15% of adolescents experience an episode of major depressive disorder (MDD), and the majority do not receive treatment.^1^^,^^2^ Evidence-based therapeutic interventions for adolescents with MDD are limited in number and efficacy and often have significant risks, including concerns about worsening depression and increased suicidality.^3^, ^4^, ^5^, ^6^ At least 40% of adolescents treated for MDD do not benefit from standard interventions.^5^^,^^7^ Barriers to effective treatment include a shortage of child and adolescent psychiatrists, limited treatment modalities, lack of precision approaches to staging treatments, health care disparities, and psychosocial factors.^8^ Neuromodulation therapies, such as transcranial magnetic stimulation (TMS), are an emerging option that may address current challenges by offering evidence-based, nonpharmacological, biological interventions for adolescents with MDD with minimal risk of significant side effects.^9^

Research on the use of TMS for adolescent MDD developed slowly after US Food and Drug Administration (FDA) clearance for adult patients in 2008.^10^, ^11^, ^12^, ^13^, ^14^, ^15^ These trials demonstrated that TMS was acceptable, well tolerated, and safe in adolescents, causing mild, transient side effects, similar to those experienced in adult samples.^16^^,^^17^ The safety of TMS in adolescents is particularly important owing to the FDA boxed warnings associated with antidepressant medications in this population. However, early prospective studies generally had small sample sizes and open-label, observational designs.^13^^,^^14^^,^^18^^,^^19^ A randomized, double-blind, sham-controlled TMS trial was conducted in 103 medication-free adolescents and young adults with MDD (ages 12-21 years); response rates of the active and sham groups were not statistically different, with rates of 41.7% and 36.4%, respectively.^20^ Nonspecific treatment effects and elevated placebo/sham response rates are well-known challenges in therapeutic trials of adolescents with MDD,^21^^,^^22^ and the relatively high rate of improvement in the sham TMS group may have obscured detection of a differential treatment effect in this trial. Furthermore, there were inherent challenges with the recruitment of adolescent subjects in this early TMS trial as antidepressant medications were withdrawn for a sham-controlled trial requiring daily visits over a period of several weeks. These requirements mirrored the design of the original pivotal trials of TMS for adults with MDD, which were conducted in medication-free patients. However, multiple, randomized controlled trials in adolescents concurrently treated with antidepressant medication suggest that TMS treatment is superior in efficacy to sham TMS or medication alone.^23^, ^24^, ^25^, ^26^

Outcome studies from patient registry databases can provide evidence of real-world effectiveness in patients receiving TMS in routine clinical practice.^27^, ^28^, ^29^, ^30^ In March 2024, the FDA revised the labeling for the NeuroStar Advanced TMS Therapy System (NeuroStar, Malvern, Pennsylvania) as an adjunctive treatment of MDD, lowering the minimum age from 22 to 15 years.^31^ The present study comprises the data that supported this FDA clearance and documents clinical outcomes in a sample of adolescents (ages 12-19 years) and young adults (ages 20-21 years) with MDD who were treated with adjunctive TMS—the largest reported sample of its type to date to our knowledge. The cutoff age of 19 years for inclusion in the adolescent group corresponds to common definitions of adolescence, including that by the World Health Organization.^32^ The adolescent and young adult samples were similar in size, so comparison of their outcomes allows for internal replication of key findings, testing the consistency of clinical effects across this age range.

We hypothesized that TMS for MDD is safe and tolerable and leads to clinically meaningful improvement in depressive symptoms in both adolescents and young adults. To evaluate safety, we examined whether a significant number of adolescents or young adults had clinically meaningful symptomatic worsening. With regard to effectiveness, we hypothesized that response and remission rates in the adolescent and young adult samples would be comparable and similar to rates previously demonstrated in the large registry sample of adults with MDD treated with TMS.

Previous research in adults shows that the extent of post-TMS improvement with once-daily treatment is contingent on the number of sessions administered; greater reduction of MDD symptoms correlates with increasing number of sessions.^30^ TMS research in adults has also demonstrated a prototypic trajectory of improvement, with rapid symptom reduction over the first 10 sessions (about 3% improvement per session) followed by a steady but considerably reduced rate of improvement (about 1% improvement per session) maintained until the end of the TMS course.^30^ In the present study, we also examined the extent to which adolescent and young adult samples are similar to adult samples in the dynamics of clinical improvement, ie, the dependence of effectiveness on number of sessions and the trajectory of symptom change during the treatment course. The primary analyses examined symptom change based on the Patient Health Questionnaire-9 (PHQ-9)^33^ self-report scores. In a considerably smaller sample, effectiveness was also examined based on the clinician-rated Clinical Global Impression-Severity (CGI-S) scale.^34^

## Method

### Clinical Outcomes Database

A retrospective analysis was conducted of open-label, naturalistic treatment data collected from the prospectively maintained NeuroStar TrakStar Clinical Database. TrakStar is a secure online database system that automatically captures deidentified patient treatment parameters, with patient demographics and clinical outcome data manually entered at the treatment sites. From November 1, 2008, to May 1, 2023, TrakStar contained data from 1,279 US sites on 115,853 unique patients who received at least 1 treatment session using the NeuroStar Advanced Therapy System Device.^27^

As a retrospective analysis of deidentified data from an existing central database, this study was conducted under an Institutional Review Board Waiver of Authorization approval, and patient privacy was maintained in compliance with the Health Insurance Portability and Accountability Act of 1996 (HIPAA). The retrospective study is registered at clinicaltrial.gov (NCT06699940).

### Participants

**Figure 1:**
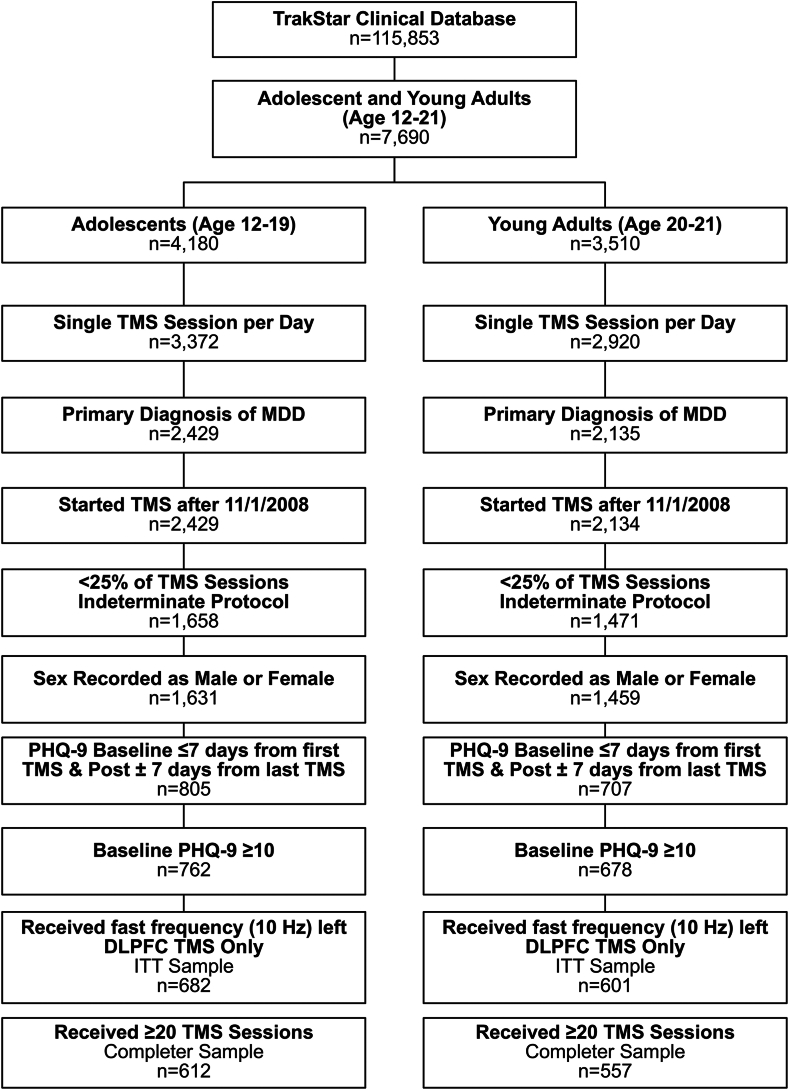
Transparent Reporting of Evaluations With Nonrandomized Designs (TREND) Diagram ***Note:****TREND diagram of study inclusion and exclusion criteria determining composition of the adolescent and young adult samples. DLPFC = dorsolateral prefrontal cortex; ITT = intent-to-treat; MDD = major depressive disorder; PHQ-9 = Patient Health Questionnaire-9; TMS = transcranial magnetic stimulation.*

In the TrakStar database, 7,690 adolescent and young adult patients (ages 12-21 years) were identified who received at least 1 TMS treatment (6.6% of the 115,853 unique patients); see Figure 1 for the Transparent Reporting of Evaluations with Nonrandomized Designs (TREND) diagram detailing sample selection. Of this group, 4,180 patients were adolescents (ages 12-19 years), and 3,510 patients were young adults (ages 20-21 years) at the start of their treatment course. To be included in the analyses, patients were required to have received all of their TMS sessions on separate days; a primary diagnosis of MDD (according to *DSM-IV-TR*/*ICD-9* or *DSM-5*/*ICD-10* criteria depending on site and date of treatment); started their treatment course on or after November 1, 2008; at least 75% of the treatment sessions in their course defined as standard, Dash, theta burst stimulation (TBS), or OCD protocol; sex listed as male or female; PHQ-9 scores available at both pre-TMS baseline (on or ≤7 days before first session) and the completion of their treatment course (±7 days from the last session of their course); and baseline PHQ-9 score ≥10, consistent with moderate to severe depression severity. The TMS course was considered complete when there was a gap in treatment >14 days after the last session. All of the above-mentioned criteria were met by 682 adolescents and 601 young adults, who were treated with left dorsolateral prefrontal cortex (DLPFC) high-frequency stimulation for all treatment sessions. This group was defined as the intent-to-treat (ITT) sample. Finally, the completer sample was defined as patients in this group who received at least 20 TMS sessions (n = 612 adolescents and n = 557 young adults). This threshold number of treatment sessions to characterize completion is consistent with the adult literature.^27^ TMS was considered off-label treatment for these patients, as it occurred before the clearance by the FDA of the expanded age range.

The patients excluded or included in the study sample were compared in age and the distributions of gender and rates of TMS completion (≥20 TMS sessions) separately for the adolescent and young adult subgroups (Table S1, available online). Representation of girls and women did not differ for excluded or included individuals. The adolescents included in the sample were significantly older than excluded adolescents, but the average difference in age was only 0.3 year; age did not distinguish the excluded and included young adults. In each subgroup, more than 80% were considered TMS completers, and rate of completion was higher in patients included in the sample. The age distributions of the overall sample and the adolescent sample were skewed toward older patients (Figure S1, available online).

### TMS Procedures

TrakStar software passively acquired information on session date, stimulation target location (ie, left DLPFC, right DLPFC, or both), motor threshold (MT) level, number of pulses prescribed or delivered per treatment location or session, stimulation intensity (ie, treatment level, percentage of device output relative to MT), pulse frequency, train duration, intertrain interval (ITI), number of trains, and number of acute phase treatment sessions. Information regarding prior or concomitant pharmacological or psychotherapeutic treatment was not obtained. Given the off-label use and FDA clearance of TMS in adults explicitly for treatment-resistant MDD, it was likely that the sample largely comprised patients receiving adjunctive TMS in private practice settings for treatment-resistant MDD.

MT was assessed at the first session with single-pulse TMS delivered over the motor cortex area corresponding with the abductor pollicis brevis muscle. The MT level was defined as the minimum device power that induced an observable thumb twitch to 50% of delivered pulses. An iterative algorithm (MT assist) facilitated this determination. MT was expressed in standardized MT units, with a standardized MT of 1.0 corresponding to the average MT level observed in a large patient sample (NeuroStar System Instructions for Use, Rev. F, Apr. 2019) and reflects an estimated electric field of 135 V/m at a point located 2.0 cm along the central axis of the treatment coil from the surface of the scalp into the patient’s cortex.

The NeuroStar stimulator had external coordinate landmarks for reproducible coil placement. Treatment target location was based either on the site on a left superior oblique plane 5.5 cm anterior to MT localization or on the site determined by standard scalp-based measurement for the F3 target.^35^ TMS protocols were classified as a function of the parameters used in the majority of sessions. All patients received left DLPFC stimulation, and the vast majority were treated with 120% MT stimulation at 10 Hz using 4-second trains, with a total of 75 trains resulting in 3,000 pulses per session. The ITI was either the classic 26 seconds,^36^ producing 37.5-minute sessions, or the newer Dash option, with ITIs ranging from 11 to 25 seconds and 18.75 minutes as the minimum session duration.^28^ A small number of patients (n = 9) were treated with an intermittent TBS protocol. The percentages of sessions each patient received the classic 26-second ITI, Dash ITI, and intermittent TBS protocols were calculated.

### Assessments

The PHQ-9^33^^,^^37^ provided the primary outcome measures, and its completion at both pre-TMS baseline and the end of the acute course was an inclusion criterion. In most patients, the PHQ-9 was completed on a weekly basis throughout the TMS course. CGI-S ratings were available in a small subset of patients.

### Statistical Analysis

Parallel analyses were conducted in the ITT and completer samples. For each sample, descriptive statistics are reported for all participants and separately for the adolescent and young adult groups. For continuous variables, mean ± SD values are presented in the tables, and mean ± SE values are presented in the figures. Sample percentages are reported for categorical variables. The ITT sample included all patients who received at least 1 TMS treatment, and the completer sample comprised all patients who received at least 20 TMS sessions, in line with the conventions in the adult literature.^27^^,^^38^ For statistical comparisons of demographic characteristics, diagnostic subtypes, TMS parameters, and symptom severity and change measures in the adolescent and young adult groups, independent sample t tests (with degrees of freedom adjusted for unequal variance) were applied for continuous variables, and Pearson χ^2^ tests were applied for categorical variables.

Dependent measures included symptom severity at baseline and the end of acute TMS; change in these scores, Pre−Post; percentage change in these scores, $\frac{\left(\text{Pre}-\text{Post}\right)\;}{\text{Pre}}\times 100$; and rates of response and remission. Response was defined as an improvement of at least 50% in the percentage change in PHQ-9 scores. Remission was defined as an end of acute TMS score less than 5.

A previous study analyzing a registry sample of 7,215 adult patients treated for MDD with TMS found a strong curvilinear association between number of sessions completed and clinical outcomes.^30^ Receiving less than 30 sessions was associated with reduced effectiveness, and clinical improvement was greatest in the group receiving 36 sessions. Patients who received extended treatment courses (>36 sessions) showed superior benefit compared with patients who received less than 30 sessions. In addition, although the extended treatment group did not attain the level of improvement seen in patients who stopped TMS after 36 sessions, they showed continued improvement beyond 36 treatments. The association between number of treatment sessions and effectiveness was similarly examined in the adolescent and young adult samples. As in the study of adults,^30^ total number of sessions comprising the treatment course was categorized into 6 groupings: 1 to 19 sessions (adolescents, n = 70; young adults, n = 44), 20 to 29 sessions (adolescents, n = 57; young adults, n = 45), 30 to 35 sessions (adolescents, n = 74; young adults, n = 88), 36 sessions (adolescents, n = 443; young adults, n = 383), 37 to 41 sessions (adolescents, n = 21; young adults, n = 31), and greater than 41 sessions (adolescents, n = 17; young adults, n = 10). An analysis of variance (ANOVA) was conducted on the percentage change in PHQ-9 scores over the TMS course, testing the main effects of age group (adolescent vs young adult) and session number group (6 levels) and the interaction between age and session number groups. To identify the factors associated with rates of response and remission, the same main effects and interactions were tested in logistic regression analyses with likelihood ratio χ^2^ tests. Post hoc pairwise comparisons of the session number groups used t tests on the percentage change in PHQ-9 scores and pairwise odds ratios for response and remission classifications using normal approximations to determine CIs.

The trajectory of symptom improvement was characterized with methods similar to those used in the large adult registry sample.^30^ The last PHQ-9 assessment completed at a time point within each of 6 treatment intervals was identified: sessions 6 to 10, sessions 16 to 20, sessions 26 to 30, sessions 32 to 36, and >36 sessions. These time points were labeled as 10 TMS, 20 TMS, 30 TMS, 36 TMS, and TMS end (end of treatment for patients receiving >36 sessions). Percent change in PHQ-9 was calculated as the difference in PHQ-9 total scores between 2 time points (T1, T2) as $\frac{\left(T1-T2\right)\;}{T1}\times 100$. Incremental rate of clinical change (typically improvement) at each time point was calculated for each patient by computing the delta in PHQ-9 percent change since the previous interval divided by the number of intervening sessions. The rates of improvement per session as a function of time point were plotted separately for each of the session number groups and for the adolescent and young adult samples.

In the total sample, the majority of patients received exactly 36 TMS sessions (n = 826, 64.4%), and 573 of these patients (adolescents, n = 306; young adults, n = 267) completed the PHQ-9 at all 4 intervals, 10 TMS, 20 TMS, 30 TMS, and 36 TMS. To test the statistical significance of changes in trajectory over the TMS course in the subsample with complete datasets, a repeated measures ANOVA was conducted on rates of PHQ-9 improvement per session with age group (adolescents or young adults) as a between-subjects factor and time point (4 levels) as the repeated measures factor. Paired t tests on rate of improvement per session were used to contrast the time point intervals.

We determined the percentage of patients with PHQ-9 score changes indicating meaningful symptomatic worsening based on the meaningful change threshold metric. The meaningful change threshold characterizes the amount of change in a measure, reflecting improvement or deterioration, that is experienced on a person-by-person basis as a meaningful change.^39^, ^40^, ^41^ In a recent reanalysis of 2 placebo controlled trials of esketamine in adults with TRD, based on Clinical Global Impression-Severity (CGI-S) anchors, Hudgens *et al.*^42^ derived the meaningful change threshold for the PHQ-9 as a change of ±6 points. Patients were classified as having a positive (≥+6 points), negative (≤−6 points), or no (≥−5 and ≤5) meaningful change based on the difference between baseline and post-TMS scores. These outcomes were computed separately for the adolescent and young adult groups, and the distributions were compared with χ^2^ analysis.

A small subgroup (n = 78; adolescents, n = 42; young adults, n = 36) was also rated by clinicians on the CGI-S both at baseline and at the end of acute TMS course. The groups with (n = 78) and without (n = 1,205) CGI-S scores were compared in PHQ-9 outcomes using t tests on continuous variables and χ^2^ analyses on categorical variables to determine whether subjects with CGI-S scores were representative of the larger sample. Response and remission were defined on the post-TMS CGI-S as scores ≤3 and ≤2, respectively, and McNemar’s test was used to compare the 2 instruments in the proportion of each type of outcome.^43^

Statistical tests were 2-tailed, with an α of .05. Statistical analyses were conducted in JMP Pro v18.0.2 (JMP Statistical Discovery, Cary, North Carolina) and SAS v9.4 (SAS Institute Inc, Cary, North Carolina).

## Results

### Participant Characteristics and Treatment Parameters

Demographic characteristics, clinical subtype, and baseline PHQ-9 scores are summarized in Table 1 for the ITT and completer samples. Approximately 60% of each group were female. On average, the young adult group was approximately 2.5 years older than the adolescent group. The great majority of both groups were classified as having recurrent MDD, and this was significantly more frequent among the young adults. In both groups, MDD with psychotic features was rare. The young adult group scored significantly higher on the PHQ-9 at baseline (mean score 19.57 vs 18.95), but the difference averaged less than 1 point. At baseline, both groups had mean PHQ-9 scores reflective of moderate-to-severe MDD.

**Table 1:** Demographic Characteristics and Clinical Features of the Intent-to-Treat (ITT) and Completer Samples

ITT sample					
	Total (N = 1,283)	Adolescents (n = 682)	Young adults (n = 601)	Statistic	*p*
	**Mean** ± **SD**	**Mean** ± **SD**	**Mean** ± **SD**		
Age, y	19.22 ± 1.50	18.09 ± 1.12	20.51 ± 0.50	*t*_969.6_ = 51.09	<.0001
	**%**	**%**	**%**		
Sex, female	61.42	60.85	62.06	χ^2^_1_ = 0.20	.66
	**Mean** ± **SD**	**Mean** ± **SD**	**Mean** ± **SD**		
Baseline PHQ-9	19.24 ± 4.21	18.95 ± 4.38	19.57 ± 3.98	*t*_1279.9_ = 2.66	.008
	**%**	**%**	**%**		
Recurrent MDD	87.84	85.92	90.02	χ^2^_1_ = 5.01	.03
MDD with psychotic features	0.55	0.29	0.83	χ^2^_1_ = 1.71	.19

**Completer sample**					
	**Total (N = 1,169)**	**Adolescents (n = 612)**	**Young adults (n = 557)**	**Statistic**	***p***
	**Mean** ± **SD**	**Mean** ± **SD**	**Mean** ± **SD**		
Age, y	19.25 ± 1.49	18.10 ± 1.11	20.51 ± 0.50	*t*_867.2_ = 48.49	<.0001
	**%**	**%**	**%**		
Sex, female	60.82	60.29	61.40	χ^2^_1_ = 0.15	.70
	**Mean** ± **SD**	**Mean** ± **SD**	**Mean** ± **SD**		
Baseline PHQ-9	19.20 ± 4.21	18.85 ± 4.41	19.58 ± 3.94	*t*_1166.6_ = 2.95	.003
	**%**	**%**	**%**		
Recurrent MDD	87.43	85.46	89.59	χ^2^_1_ = 4.52	.03
MDD with psychotic features	0.60	0.33	0.90	χ^2^1 = 1.60	.21

Note: MDD = major depressive disorder; PHQ-9 = Patient Health Questionnaire-9.

As seen in Table S2 (available online), the adolescent and young adult groups differed significantly in 1 TMS treatment parameter: resting MT was lower in the young adult sample. The acute treatment course averaged about 8 weeks and 35 sessions in the completer sample. The vast majority of participants received the classic left DLPFC stimulation parameters, ie, 10 Hz, 4-second trains, 3,000 pulses per session, delivered at 120% of MT. In nearly 90% of sessions, the Dash protocol was used with an ITI of 11 seconds. The classic 26-second ITI was used in approximately 10% of sessions, and use of the intermittent TBS protocol was rare (<1%).

### PHQ-9 Antidepressant Effects

Table 2 summarizes the changes in PHQ-9 scores over the TMS course and PHQ-9 response and remission rates. The adolescent and young adult groups did not differ in final PHQ-9 score, in percentage change from baseline in this score, or in response or remission rates. The young adult group had a significantly larger arithmetic drop in these scores, ultimately eliminating the difference in symptom severity between groups that was present at baseline. In both groups, response and remission rates were substantial, averaging approximately 60% and 30%, respectively. The extent of symptomatic improvement measured by PHQ-9 and the overall response and remission rates in our samples were only slightly less than the outcomes reported in a completer sample of 2,053 adults similarly treated only with high-frequency, left DLPFC stimulation. In that registry study of adults, PHQ-9 response and remission rates were approximately 68% and 36%, respectively.

**Table 2:** PHQ-9 Treatment Outcomes in the Intent-to-Treat (ITT) and Completer Samples

ITT sample					
	Total (N = 1,283)	Adolescents (n = 682)	Young adults (n= 601)	Statistic	*p*
	**Mean** ± **SD**	**Mean** ± **SD**	**Mean** ± **SD**		
End of acute PHQ-9	9.71 ± 6.79	9.84 ± 6.79	9.56 ± 6.79	*t*_1260.5_ = 0.76	.45
Change in PHQ-9	9.53 ± 6.74	9.10 ± 6.73	10.01 ± 6.74	*t*_1260.4_ = 2.41	.02
Percent change PHQ-9	49.57 ± 33.12	48.10 ± 33.44	51.23 ± 32.71	*t*_1267.1_ = 1.69	.09
	**%**	**%**	**%**		
Response rate	55.73	53.37	58.40	χ^2^_1_ = 3.28	.07
Remission rate	28.14	27.57	28.79	χ^2^_1_ = 0.24	.63
Meaningful change threshold				χ^2^_2_ = 2.09	.35
Positive	70.85	69.21	72.71		
Neutral	28.22	29.91	26.29		
Negative	0.94	0.88	1.00		

**Completer sample**					
	**Total (N = 1,169)**	**Adolescents (n = 612)**	**Young adults (n = 557)**	**Statistic**	***p***
	**Mean** ± **SD**	**Mean** ± **SD**	**Mean** ± **SD**		
End of acute PHQ-9	9.19 ± 6.57	9.23 ± 6.52	9.14 ± 6.63	*t*_1153.0_ = 0.22	.82
Change in PHQ-9	10.01 ± 6.62	9.63 ± 6.59	10.44 ± 6.63	*t*_1155.6_ = 2.08	.04
Percent change in PHQ-9	52.14 ± 32.30	50.01 ± 32.54	53.38 ± 32.02	*t*_1159.9_ = 1.25	.21
	**%**	**%**	**%**		
Response rate	59.37	57.52	61.40	χ^2^_1_ = 1.82	.18
Remission rate	30.03	29.74	30.34	χ^2^_1_ = 0.05	.82
Meaningful change threshold				χ^2^_2_ = 1.97	.37
Positive	73.74	72.22	75.40		
Neutral	25.32	26.96	23.52		
Negative	0.94	0.82	0.94		

Note: PHQ-9 = Patient Health Questionnaire-9.

### Number of Sessions and Effectiveness

**Figure 2:**
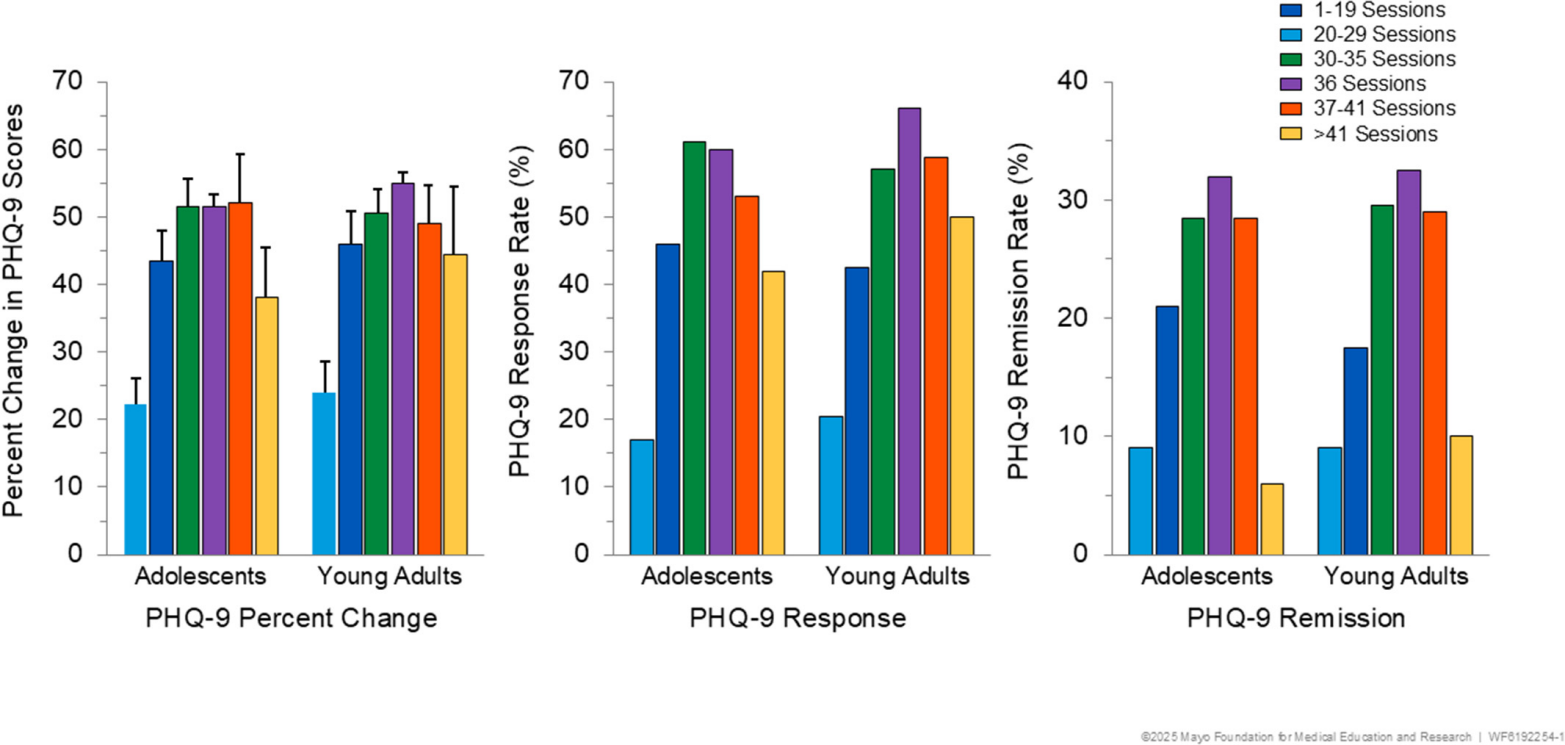
Dose-Response Relationships ***Note:****Association between total number of transcranial magnetic stimulation sessions administered and percent change in Patient Health Questionnaire-9 (PHQ-9) scores (left), response rate (middle), and remission rate (right) separately for adolescent (ages 12-19 years) and young adult (ages 20-21 years) samples with major depressive disorder. Duration of transcranial magnetic stimulation course was categorized as less than 20, 20 to 29, 30 to 35, 36, 37 to 41, and >41 sessions.*

The ANOVA conducted on the percentage change in PHQ-9 scores and the logistic regression analyses conducted on response and remission rates did not yield main effects of age group (all *p*s ≥ .54). This confirmed that, overall, the adolescent and young adult samples manifested comparable PHQ-9 antidepressant effects. As illustrated in Figure 2, there was a marked main effect of session number group on each outcome measure: percentage change in PHQ-9 scores (*F*_5,1271_ = 18.07, *p* < .0001); response rate (χ^2^_5_ = 84.71, *p* < .0001); remission rate (χ^2^_5_ = 43.01, *p* < .0001). Post hoc comparisons indicated that the patients treated with 1 to 19 sessions and, to a lesser extent, with 20 to 29 sessions had lower percentage improvement in PHQ-9 scores and lower rates of response and remission than other groups of patients who received more sessions. The groups that received 30 to 35 sessions or exactly 36 sessions had superior outcomes relative to other groups. These dose-response effects were highly similar in the adolescent and young adult samples (Figure 2) and replicated the patterns reported in a large and similarly treated adult sample.^30^ There were no significant interactions involving age group and session number group (all *p*s ≥ .84), indicating that the association between total number of sessions and clinical outcome was the same for adolescents and young adults.

### Trajectory of Symptom Improvement During TMS

**Figure 3:**
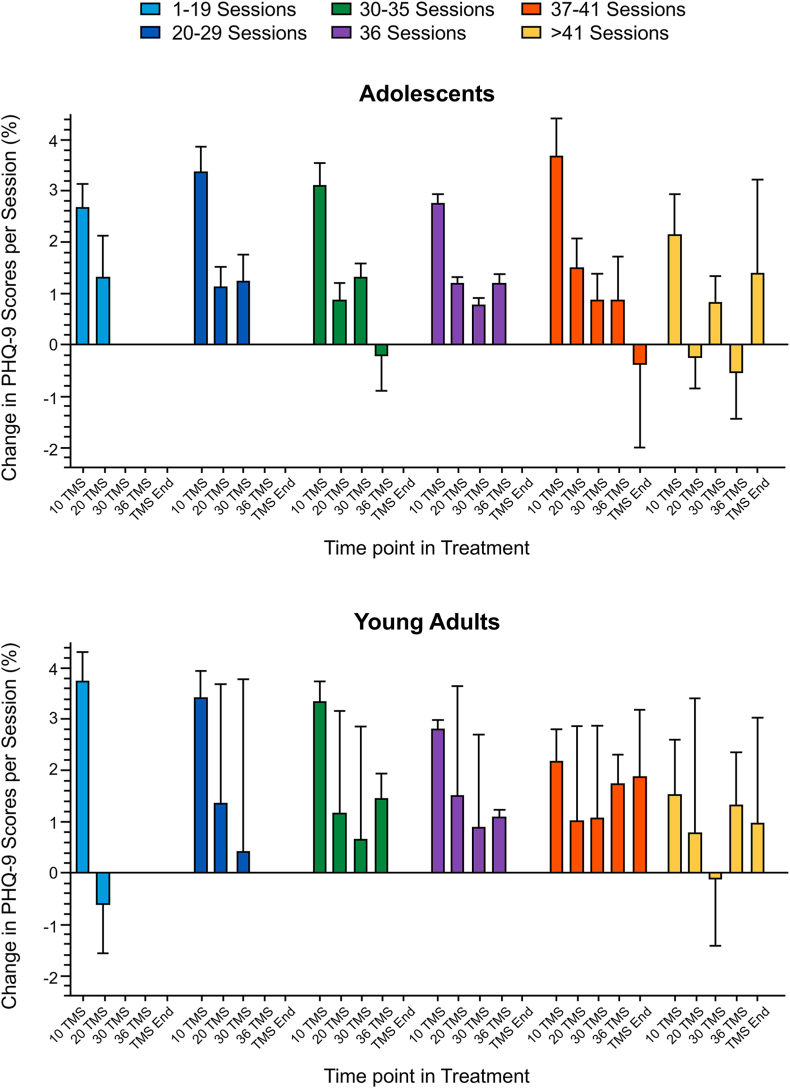
Trajectory of Improvement ***Note:****Percent improvement in Patient Health Questionnaire-9 (PHQ-9) scores per session with time point reflecting assessments after 10, 20, 30, 36, and final (>36) transcranial magnetic stimulation (TMS) sessions. These rates of improvement are plotted separately for groups that received 1 to 19, 20 to 29, 30 to 35, 36, 37 to 41, and >41 sessions and separately for the adolescent (ages 12-19 years) and young adult (ages 20-21 years) samples with major depressive disorder.*

Figure 3 presents the average improvement per session at the fixed time points separately for each of the session number groups and the adolescent and young adult samples. The repeated measures ANOVA conducted in the patients who received exactly 36 sessions yielded a significant main effect of time point (*F*_3,1713_ = 55.36, *p* < .0001) with no effect of age group (*F*_1,571_ = 0.88, *p* = .35) and no interaction between age group and time point (*F*_3,1713_ = 0.82, *p* < .58). Across the adolescent and young adult samples, paired t tests demonstrated that the percent improvement in PHQ-9 scores per session after 10 sessions (approximately 3% per session) greatly exceeded the improvement rates at all subsequent intervals (approximately 1% per session) (all *p*s < .0001). This pattern was seen in all groups that received 36 or fewer sessions. The groups that received longer courses of TMS were distinguished by a slower rate of improvement after the first 10 sessions. These patterns are highly similar to trajectories of improvement observed in the large adult registry sample.^30^

### Safety: Worsening of Depressive Symptoms

Table 2 presents the distributions of individuals with a positive, no, or negative meaningful change in PHQ-9 scores following TMS. The adolescent and young adult samples did not differ in these distributions. More than 70% of the entire sample experienced a positive meaningful change, whereas less than 1% experienced a negative meaningful change. Thus, clinically meaningful worsening of depressive symptoms was rare.

### Clinician-Rated (CGI-S) Antidepressant Effects

As seen in Table S3 (available online), patients who did not have CGI-S ratings scored higher on the PHQ-9 at baseline than patients with CGI-S ratings. However, the 2 groups did not differ in the change or percentage change in PHQ-9 scores over the TMS course or in PHQ-9 response or remission rates. The adolescent and young adult groups did not differ in any CGI-S measure (Table S4, available online). Across all patients with CGI-S ratings, rates of response were substantially greater on the CGI-S than when assessed with the PHQ-9 (76.9% vs 57.7%, McNemar χ^2^_1_ = 15.0, *p* < .0001); this was also true for rates of remission (47.4% vs 29.5%, McNemar χ^2^_1_ = 9.8, *p* = .002). In the completer sample (n = 77), 77.9% of patients had CGI-S ratings commensurate with response, and 48.1% attained remission.

## Discussion

This observational study examined the largest sample to date of adolescents and young adults treated with TMS for MDD. In both the adolescent and the young adult samples, the overall magnitude of clinical improvement, trajectory of improvement, and dose-response relationship with number of treatment sessions replicated the same effects observed in adult MDD.^27^^,^^30^ More than 70% of adolescent and young adult patients demonstrated meaningful positive improvement in depression symptoms, whereas fewer than 1% had a meaningful negative change. Symptom reduction was strongly correlated with the number of treatment sessions and did not plateau at treatment termination. These findings supported the recent clearance of TMS by the FDA as an adjunctive treatment of MDD in adolescent and young adult patients (ages 15-21 years).^44^

Systematic reviews have consistently supported the safety and effectiveness of TMS in adolescent MDD^45^, ^46^, ^47^ and included findings that TMS effectiveness compares favorably with electroconvulsive therapy.^48^ A recent meta-analysis of 6 randomized trials conducted in China in adolescents with first-episode MDD compared TMS plus antidepressant medication with either antidepressant alone or antidepressant combined with sham TMS. The results indicated that adjunctive TMS was markedly superior in antidepressant effects relative to control conditions.^26^ In contrast, in a multicenter randomized controlled trial, monotherapy with TMS did not differ from sham TMS in medication-free adolescents with treatment-resistant depression.^20^ It is a matter of speculation why this negative result was obtained, but it was consistent with multiple pharmacological trials in adolescent MDD that yielded negative findings in the context of high rates of improvement with placebo.^21^^,^^22^ In the TMS randomized controlled trial that generated negative results, participants were withdrawn from antidepressant medications a week before screening and remained off them throughout the 6 weeks of daily blinded treatment with active or sham TMS. These trial requirements may have limited the representativeness of the adolescent sample. Early TMS trials in adult samples also withdrew participants from antidepressant medications and had relatively low response and remission rates compared with more recent studies in which TMS was investigated as an adjunctive treatment.^27^^,^^36^^,^^49^

The strength of the current retrospective study was the inclusion of all adolescents and young adults with MDD treated since November 2008 with a NeuroStar device, with data generated by 364 diverse sites across the United States that obtained PHQ-9 ratings before and after the treatment course. The adolescent and young adult subgroups did not differ in any outcome measure, and comparisons between them provided internal replication of the major effects. Both age groups showed substantial reduction in PHQ-9 scores following TMS, with rates of response and remission similar to those reported in adults. The overall benefit did not seem to be an artifact of relying on self-reports of symptom severity, as clinical outcomes were stronger in the small subsample that also had clinician-based CGI-S ratings, a pattern also seen in the adult population.^27^ Indeed, both the adolescent and the young adult groups also mirrored the adult patterns in terms of the association between number of TMS sessions administered and clinical outcome and the trajectory of improvement over the TMS course.^30^ As in adults, effectiveness was greatest in the group receiving 36 sessions with substantially poorer outcome in patients who received fewer than 30 sessions. As in adults, the extent of improvement in PHQ-9 scores averaged about 3% per session over the first 10 sessions, then decreased to about 1%, and then remained stable until treatment termination. The similarities between the adolescent and the young adult groups with adults in the extent and dynamics of clinical change with TMS provide support to challenge the idea that antidepressant response to TMS in adolescents solely reflects a placebo effect. Whereas one could argue that placebo effects are a major driver of response to every treatment available for MDD in every population, multiple sham-controlled^36^^,^^49^ and comparator trials^50^ have established the efficacy of TMS in adults.

This study had important limitations. The adolescents in the sample were predominantly older (ages 17-19 years), and this is a consideration for interpreting the findings in the context of clinical practice. The only data included in the deidentified database were basic demographics (age and sex at birth), diagnostic status, treatment parameters, and depression inventory scores. There was no information on clinical or treatment history or concurrent pharmacological treatments. The outcome data were based on self-report with an instrument assessing limited symptom domains, and there is a need for validation against clinician ratings and more thorough evaluation of phenomenology, particularly suicidality. CGI-S scores were available in only a small portion of the sample. However, recent work suggests that the PHQ-9 may have utility in evaluating the antidepressant effects of TMS treatments.^51^ Furthermore, TrakStar software did not capture adverse events or side effects, and the only safety data examined here were the rate of meaningful symptomatic worsening in PHQ-9 scores. The dataset did not include scores on individual PHQ-9 items, and this limited our ability to examine improvements or worsening in suicidal ideation over the course of treatment. The sample was likely limited with respect to socioeconomic diversity, especially given the off-label use of TMS. This should be addressed in future work. The database focused on daily TMS treatments with 1 particular device with a figure-of-eight coil. TMS devices with figure-of-eight coils have previously been shown to be equivalent, so it is expected that the present findings would generalize to other TMS devices with similar coils with protocols delivering single daily sessions of TMS. There are open questions regarding the effects of deep TMS protocols, accelerated TMS protocols, TMS treatments for obsessive-compulsive disorder, and TMS treatments for smoking cessation in adolescents that merit further prospective study.^27^, ^28^, ^29^, ^30^, ^31^

Adjunctive TMS is now an FDA-cleared treatment modality for adolescents and young adults with MDD, including individuals who have not failed prior antidepressant therapy. This study documented that a large and geographically diverse sample of adolescents and young adults with MDD had clinically meaningful improvement in symptom severity with adjunctive TMS treatment. Rates of response and remission, trajectories of depressive symptom improvement, and the association of session number with effectiveness were congruent with the patterns in adults. Future research should focus on investigating treating to remission rather than a predefined number of sessions and accelerating symptomatic improvement. Future studies of TMS for the treatment of MDD in adolescents should focus on predictors of response and remission, durability of benefit, and comparative effectiveness when compared with an additional medication trial.

## CRediT authorship contribution statement

**Paul E. Croarkin:** Writing – review & editing, Writing – original draft, Visualization, Validation, Supervision, Project administration, Methodology, Investigation, Formal analysis, Data curation, Conceptualization. **Scott T. Aaronson:** Writing – review & editing, Visualization, Validation, Methodology, Investigation, Formal analysis, Data curation, Conceptualization. **Linda L. Carpenter:** Writing – review & editing, Visualization, Validation, Methodology, Investigation, Formal analysis, Data curation, Conceptualization. **Todd M. Hutton:** Writing – review & editing, Visualization, Validation, Investigation, Formal analysis, Data curation, Conceptualization. **Kenneth Pages:** Writing – review & editing, Visualization, Validation, Investigation, Data curation, Conceptualization. **Bing Chen:** Writing – review & editing, Visualization, Validation, Formal analysis, Conceptualization. **Harold A. Sackeim:** Writing – review & editing, Writing – original draft, Visualization, Validation, Supervision, Project administration, Methodology, Investigation, Formal analysis, Data curation, Conceptualization.
