# Supplementary figures and images for: The Effectiveness of Transcranial Magnetic Stimulation in Adolescents and Young Adults With Major Depressive Disorder
Source: JAACAP Open. 2025 Jul 1;3(4):1246–58. doi: 10.1016/j.jaacop.2025.06.006 (PMC12684655; doi:10.1016/j.jaacop.2025.06.006)

# Age Distribution of Study Participants (N=1283)

| Age | Number of Participants |
|-----|------------------------|
| 12  | 2                      |
| 13  | 2                      |
| 14  | 7                      |
| 15  | 17                     |
| 16  | 31                     |
| 17  | 61                     |
| 18  | 280                    |
| 19  | 282                    |
| 20  | 297                    |
| 21  | 304                    |

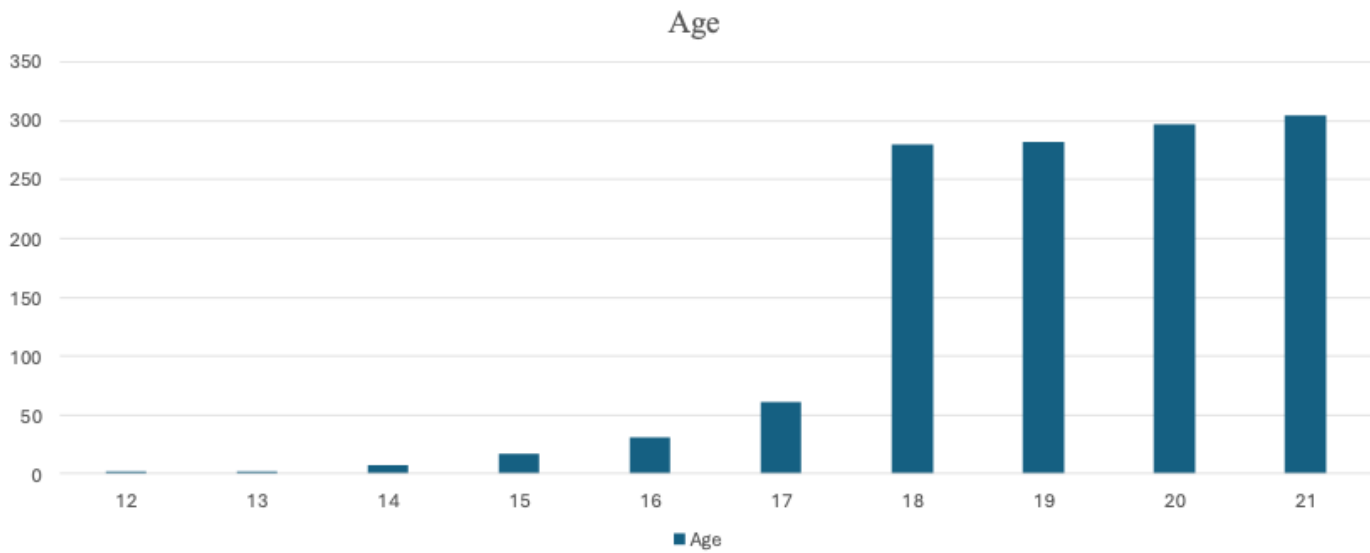

Supplement: Supplementary Figure S1 [file mmc1.pdf]
