# Supplementary material for: The Effectiveness of Transcranial Magnetic Stimulation in Adolescents and Young Adults With Major Depressive Disorder
Source: JAACAP Open. 2025 Jul 1;3(4):1246–58. doi: 10.1016/j.jaacop.2025.06.006 (PMC12684655; doi:10.1016/j.jaacop.2025.06.006)
Supplement: Supplementary Tables [file mmc2.docx]

Supplementary Table 1. Demographic features and TMS completer rates for patients excluded or included in the study sample, separately for adolescent and young adult subgroups.

|  | **Adolescent (age 12-19 yr)** | | |  | |
| --- | --- | --- | --- | --- | --- |
|  | **Excluded from sample** | **Included  in sample** |  | |  |
|  | **N = 3498** | **N= 682** | **Statistic** | | **P** |
| Age (yr) | 17.79±1.41 | 18.09 ± 1.12 | *t*(1146.4) = 5.99 | | <0.0001 |
| Sex (% female)* | 61.22 | 60.85 | *X*^2^(1) = 0.03 | | 0.86 |
| Completer (≥ 20 TMS sessions) (%) | 82.10 | 89.74 | *X* ^2^(1) = 23.95 | | <0.0001 |
|  | **Young Adult (age 20-21 yr)** | | |  | |
|  | **Excluded from sample** | **Included in sample** |  | |  |
|  | **N = 2909** | **N= 601** | **Statistic** | | **P** |
| Age (yr) | 20.51 ± 0.50 | 20.51 ± 0.50 | *t*(865.5) = 0.88 | | 0.88 |
| Sex (% female)* | 57.91 | 62.06 | *X* ^2^(1) = 3.54 | | 0.06 |
| Completer (≥ 20 TMS sessions) (%) | 80.47 | 92.68 | *X* ^2^(1) = 51.54 | | <0.0001 |

* Percent female rates do not include individuals coded as neither male or female which was an exclusion criterion. Among the adolescent subgroup, 48 individuals were coded as neither male or female and this applied to 27 individual sessions the young adult subgroup.

Supplementary Table 2. TMS Treatment Parameters in the Intent-to-Treat and Completer Samples

|  |  | **ITT Sample** |  |  | |  | |
| --- | --- | --- | --- | --- | --- | --- | --- |
|  | **Total** | **Adolescents** | **Young Adults** |  | |  | |
|  | **N = 1283** | **N = 682** | **N= 601** | **Statistic** | | **P** | |
| Total Sessions in TMS Course | 32.83 ± 7.96 | 32.56 ± 8.27 | 33.13 ± 7.59 | *t*(1279.0) = 1.29 | | 0.20 | |
| Days in TMS Course | 54.66 ± 15.84 | 54.11 ± 16.22 | 55.29 ±15.40 | *t*(1273.9) = 1.33 | | 0.18 | |
| Motor Threshold (SMT Unit) | 1.02 ± 0.24 | 1.03 ± 0.24 | 1.01 ± 0.24 | *t*(1265.9) = 1.78 | | 0.08 | |
| Treatment Intensity (% MT) | 117.04 ± 9.95 | 116.70 ± 10.24 | 117.44 ± 9.60 | *t*(1276.1) = 1.34 | | 0.18 | |
| Pulse Frequency* (Hz) | 10.15 ± 1.32 | 10.12 ± 1.29 | 10.18 ± 1.36 | *t*(1231.6) = 0.82 | | 0.41 | |
| Train Duration (s) | 4.00 ± 0.22 | 3.99 ± 0.24 | 4.00 ± 0.20 | *t*(1276.3) = 0.35 | | 0.73 | |
| Inter-train Interval (ITI, s) | 13.48 ± 5.26 | 13.38 ± 5.20 | 13.59 ± 5.32 | *t*(1253.6) = 0.71 | | 0.48 | |
| Pulses per Session | 2981.68 ± 207.05 | 2979.18 ± 221.48 | 2984.53 ± 189.50 | *t*(1279.9) = 0.47 | | 0.64 | |
| Protocol (% of sessions) |  |  |  |  | |  | |
| 10 Hz (26 s ITI) | 10.98 ± 29.09 | 10.55 ± 28.34 | 11.41 ± 29.94 | *t*(1240.3) = 0.53 | | 0.60 | |
| 10 Hz (shortened ITI) | 87.46 ± 30.22 | 87.84 ± 29.52 | 87.03 ± 31.01 | *t*(1242.7) = 0.48 | | 0.63 | |
| iTBS | 0.88 ± 8.32 | 0.82 ± 8.08 | 0.95 ± 8.58 | *t*(1237.6) = 0.27 | | 0.79 | |
|  |  |  |  |  | |  | |
|  |  | **Completer Sample** | |  | |  | |
|  | **Total** | **Adolescents** | **Young Adult** |  | |  | |
|  | **N = 1169** | **N = 612** | **N= 557** | **Statistic** | | **P** | |
| Total Sessions in TMS Course | 34.92 ± 4.22 | 34.95 ± 4.21 | 34.89 ± 4.23 | *t*(1155.5) = 0.25 | | 0.80 | |
| Days in TMS Course | 58.18 ± 11.23 | 58.14 ± 11.14 | 58.23 ± 11.34 | *t*(1152.6) = 0.14 | | 0.89 | |
| Motor Threshold (SMT Unit) | 1.02 ± 0.24 | 1.03 ± 0.24 | 1.00 ± 0.23 | *t*(1160.8) = 2.01 | | 0.045 | |
| Treatment Intensity (% MT) | 118.03 ± 7.76 | 117.84 ± 7.94 | 118.24 ± 7.56 | *t*(1164.6) = 0.88 | | 0.38 | |
| Pulse Frequency* (Hz) | 10.16 ± 1.32 | 10.14 ± 1.31 | 10.18 ± 1.33 | *t*(1164.6) = 0.49 | | 0.63 | |
| Train Duration (s) | 4.00 ± 0.20 | 4.00 ± 0.20 | 4.00 ± 0.20 | *t*(1144.0) = 0.49 | | 0.63 | |
| Inter-train Interval (ITI, s) | 13.50 ± 5.29 | 13.39 ± 5.21 | 13.62 ± 5.38 | *t*(1148.5) = 0.76 | | 0.45 | |
| Pulses per Session | 2984.09 ± 192.06 | 2984.80 ± 187.80 | 2983.30 ± 196.80 | *t*(1144.3) = 0.13 | | 0.89 | |
| Protocol (% of sessions) |  |  |  |  |  | |  |
| 10 Hz (26 s ITI) | 11.01 ± 29.15 | 10.35 ± 28.12 | 11.73 ± 30.26 | *t*(1135.3) = 0.80 | 0.42 | |  |
| 10 Hz (shortened ITI) | 87.44 ± 30.14 | 88.20 ± 28.96 | 86.61 ± 31.38 | *t*(1132.6) = 0.90 | 0.37 | |  |
| iTBS | 0.90 ± 8.45 | 0.80 ± 8.02 | 1.02 ± 8.91 | *t*(1122.5) = 0.46 | 0.65 | |  |

*Excludes 9 ITT sample participants and 8 Completer sample participants who were treated with iTBS only.

Supplementary Table 3. Change in PHQ-9 Scores and Response and Remission Rates in those with and without CGI-S Scores

|  | **Total** | **Without CGI-S** | **With CGI-S** |  |  |
| --- | --- | --- | --- | --- | --- |
|  | **N = 1283** | **N = 1205** | **N= 78** | **Statistic** | **P** |
| Baseline PHQ-9 | 19.24 ± 4.21 | 19.31 ± 4.20 | 18.15 ± 4.16 | *t*(87.5) = 2.37 | 0.008 |
| EOA PHQ-9 | 9.71 ± 6.79 | 9.77 ± 6.77 | 8.73 ± 6.79 | *t*(86.7) = 1.28 | 0.20 |
| Change in PHQ-9 | 9.53 ± 6.74 | 9.54 ± 6.73 | 9.42 ± 6.98 | *t*(86.5) = 0.14 | 0.87 |
| Percentage Change in PHQ-9 | 49.57 ± 33.12 | 49.43 ± 32.88 | 51.70 ± 36.76 | *t*(85.2) = 0.53 | 0.60 |
| Response Rate (%) | 55.73 | 55.60 | 57.69 | *X*^2^(1) = 0.13 | 0.72 |
| Remission Rate (%) | 28.14 | 28.05 | 29.49 | *X*^2^(1) = 0.08 | 0.78 |

Supplementary Table 4. Change in CGI-S Scores and Response and Remission Rates in the Intent-to-Treat Sample

|  | **Total** | **Adolescents** | **Young Adults** |  |  |
| --- | --- | --- | --- | --- | --- |
|  | **N = 78** | **N = 42** | **N= 36** | **Statistic** | **P** |
| Baseline CGI-S | 5.33 ± 0.83 | 5.26 ± 0.80 | 5.42 ± 0.87 | *t*(71.7) = 0.81 | 0.42 |
| EOA CGI-S | 2.69 ± 1.33 | 2.79 ± 1.24 | 2.58 ± 1.44 | *t*(69.6) = 0.66 | 0.51 |
| Change in CGI-S | 2.64 ± 1.28 | 2.48 ± 1.17 | 2.83 ± 1.38 | *t*(69.1) = 1.22 | 0.23 |
| Percentage Change in CGI-S | 49.67 ± 22.95 | 47.34 ± 21.00 | 52.39 ± 25.06 | *t*(68.6) = 0.96 | 0.34 |
| Response Rate (%) | 76.92 | 76.19 | 77.78 | *X*^2^(1) = 0.03 | 0.87 |
| Remission Rate (%) | 47.44 | 40.48 | 55.56 | *X*^2^(1) = 1.77 | 0.18 |

*In the Completer Sample (N=77), 77.9% of subjects attained CGI-S response and 48.1% attained CGI-S remission.
